# Gait Features in Different Environments Contributing to Participation in Outdoor Activities in Old Age (GaitAge): Protocol for an Observational Cross-Sectional Study

**DOI:** 10.2196/52898

**Published:** 2024-04-29

**Authors:** Merja Rantakokko, Emmi Matikainen-Tervola, Eeva Aartolahti, Sanna Sihvonen, Julija Chichaeva, Taija Finni, Neil Cronin

**Affiliations:** 1 Faculty of Sport and Health Sciences Gerontology Research Center University of Jyväskylä Jyväskylä Finland; 2 The Wellbeing Services County of Central Finland Jyväskylä Finland; 3 Institute of Rehabilitation JAMK University of Applied Sciences Jyväskylä Finland; 4 Faculty of Sport and Health Sciences Neuromuscular Research Centre University of Jyväskylä Jyväskylä Finland; 5 School of Sport and Exercise University of Gloucestershire Gloucester United Kingdom

**Keywords:** walking, aging, environment, biomechanics, kinematics, spatiotemporal, gait, GaitAge, observational cross-sectional study, gerontology, geriatric, geriatrics, older adult, older adults, elder, elderly, older person, older people, ageing, aging, health disparities, health disparity, assessment, assessments, physical test, physical tests, interview, interviews, biomechanic, activities, outdoor, activity, movement analysis, analysis of walk, posture, free living

## Abstract

**Background:**

The ability to walk is a key issue for independent old age. Optimizing older peoples’ opportunities for an autonomous and active life and reducing health disparities requires a better understanding of how to support independent mobility in older people. With increasing age, changes in gait parameters such as step length and cadence are common and have been shown to increase the risk of mobility decline. However, gait assessments are typically based on laboratory measures, even though walking in a laboratory environment may be significantly different from walking in outdoor environments.

**Objective:**

This project will study alterations in biomechanical features of gait by comparing walking on a treadmill in a laboratory, level outdoor, and hilly outdoor environments. In addition, we will study the possible contribution of changes in gait between these environments to outdoor mobility among older people.

**Methods:**

Participants of the study were recruited through senior organizations of Central Finland and the University of the Third Age, Jyväskylä. Inclusion criteria were community-dwelling, aged 70 years and older, able to walk at least 1 km without assistive devices, able to communicate, and living in central Finland. Exclusion criteria were the use of mobility devices, severe sensory deficit (vision and hearing), memory impairment (Mini-Mental State Examination ≤23), and neurological conditions (eg, stroke, Parkinson disease, and multiple sclerosis). The study protocol included 2 research visits. First, indoor measurements were conducted, including interviews (participation, health, and demographics), physical performance tests (short physical performance battery and Timed Up and Go), and motion analysis on a treadmill in the laboratory (3D Vicon and next-generation inertial measurement units [NGIMUs]). Second, outdoor walking tests were conducted, including walking on level (sports track) and hilly (uphill and downhill) terrain, while movement was monitored via NGIMUs, pressure insoles, heart rate, and video data.

**Results:**

A total of 40 people (n=26, 65% women; mean age 76.3, SD 5.45 years) met the inclusion criteria and took part in the study. Data collection took place between May and September 2022. The first result is expected to be published in the spring of 2024.

**Conclusions:**

This multidisciplinary study will provide new scientific knowledge about how gait biomechanics are altered in varied environments, and how this influences opportunities to participate in outdoor activities for older people.

**International Registered Report Identifier (IRRID):**

RR1-10.2196/52898

## Introduction

### Overview

The ability to walk is a key issue in maintaining independence in old age. Walking difficulties hinder the ability to manage tasks of daily life and may lead to the need for help and an increased risk of disability and institutionalization [1,2]. With increasing age, changes in gait are common [3]. Walking speed declines with increasing age [4], due to changes in step or stride length, as well as slower cadence. Changes in ankle, knee, and hip motion and center of pressure are also common [4,5]. Gait variability, defined as fluctuations in spatiotemporal characteristics between steps increases with age [3,6] and is associated with increasing risk of developing mobility difficulties [7].

Spatiotemporal and kinematic parameters of gait provide important information, but outcomes are usually based on a treadmill or laboratory overground gait, which may be significantly different from walking outdoors. Only a few studies have examined differences in gait biomechanics between laboratory and outdoor circumstances among older people. In the studies of Schmitt et al [6] and Renggli et al [8] significant differences in several spatiotemporal parameters were found between outdoor walking and treadmill walking. On a treadmill, a decrease in walking speed, an increase in double support duration, shorter stride length, and decreased cadence were found compared to outdoor walking [6,8]. While these assessments provided knowledge about issues such as fall prediction [9] many uncertainties remain. For example, the environment where the activity takes place is typically not recorded, so it is not known whether changes in gait represent a response to some specific environmental demand or whether it is an expression of a person’s usual performance. Taking into account environmental features, and using wearable sensors, such as inertial measurement units (IMUs) at the same time could add important information about walking speed and body orientation that is needed to understand how people move and adapt to challenges encountered in different environmental circumstances.

The ability to adapt gait to environmental demands may be crucial for preventing or reducing restrictions in participation on outdoor activities. The concept of life-space mobility gives us an idea about how well people are able to access different community amenities, attend different events, or be physically active, thus describing a person’s opportunities for participation outside the home [10]. Life-space mobility refers to the spatial area in which a person moves in daily life, taking into account distance, frequency, and any assistance needed for movement. Restrictions in participating in out-of-home activities are known to correlate, for example, with functional limitations, pain, depression, and chronic conditions [11-13]. Previously it has also been shown that life-space mobility correlates with, for example, quality of life [14,15], physical functioning, and autonomy [16], and that restrictions on it may lead to cognitive decline [17,18], falls and fractures [19], nursing home admission [20], or even mortality [21]. However, research on the association between gait parameters and the impact on life-space mobility is scarce.

### Study Objectives

The aim of the “Gait features in different environments contributing to participation in outdoor activities in old Age (GaitAge)” project is to study whether IMUs are valid for detecting traditional (gait cycle event timings) and novel (heading and torso angle) gait parameters among older people in laboratory and outdoor environments. We will focus on how gait parameters change when walking on a treadmill in a laboratory compared to an outdoor environment and in a hilly environment compared to walking in a level outdoor environment. Finally, we will study whether alterations in gait parameters in different environments are associated with outdoor participation (measured via life-space mobility and physical activity) in older people when health and demographics are taken into account.

## Methods

### Recruitment

Our target was to recruit a total of 40 participants for the study. First, we invited 15 participants from our pilot study conducted between May and June 2021 (not published), who had given their permission to contact them again. These participants were recruited through the University of the Third Age in Jyväskylä by advertising in a public lecture in May 2021. They were contacted again in April 2022 to enquire about their interest in taking part in this study. All of them were willing to take part in the study and met the inclusion criteria.

Additional recruitment to reach a total of 40 participants was done in June 2022 through 5 central Finland senior organizations. Leaders of these organizations were contacted via email and were asked to forward an information letter and invitation to all members of the organizations. Those members who were interested in taking part in the study were asked to contact researchers via email or by phone, after which a short phone interview was scheduled.

A researcher called potential participants and conducted a short telephone interview to screen their suitability and to confirm their willingness to participate. Inclusion criteria were community-dwelling, at least 70 years of age during the current year, able to walk at least 1 km without assistive devices, able to communicate, and living in central Finland. Exclusion criteria were the use of mobility devices, severe sensory deficit (vision and hearing), neurological conditions (eg, stroke, Parkinson disease and multiple sclerosis), and memory impairment (Mini-Mental State Examination [MMSE]≤23) [22]. Memory impairment was assessed during the initial phone interview by asking about diagnosed memory illness and screened with an MMSE test during the first research visit to the laboratory.

Recruitment continued until 40 participants who met the inclusion criteria and were willing to participate were reached.

### Data Collection

**Figure 1:**
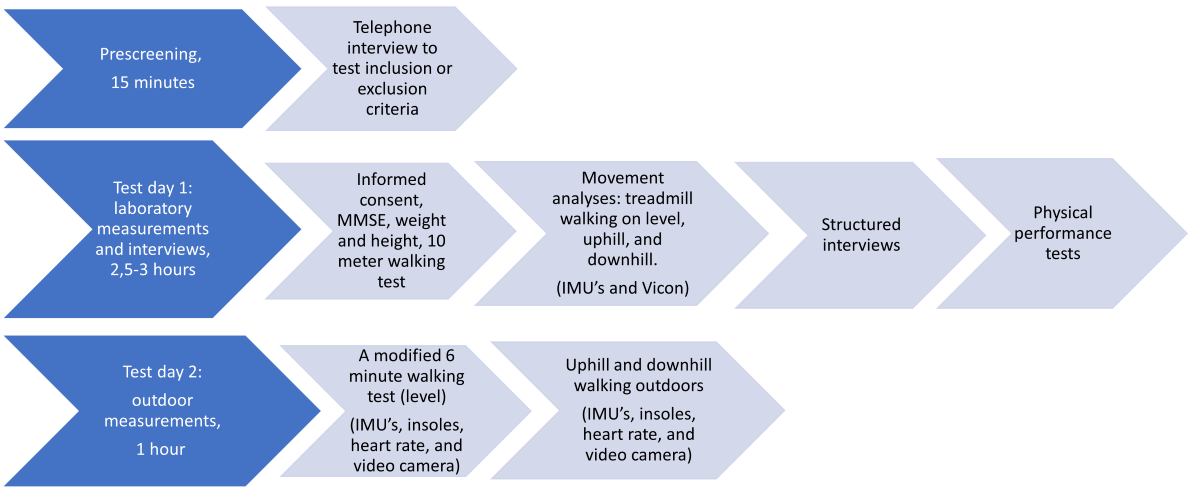
Data collection protocol of GaitAge project. IMU: Inertial measurement unit; MMSE: Mini-Mental State Examination.

The data collection procedure is shown in Figure 1. Data collection took place between May 27, 2022, and September 28, 2022. The data collection protocol included two visits (1) indoor walking measurements and interviews that were conducted at the University of Jyväskylä sport and exercise laboratory and (2) approximately 4 days later, outdoor walking measurements were conducted at the local sports track and hilly terrain next to the sports track. For some participants, the time between indoor and outdoor walking measurements was longer than 3 days due to weather conditions, as it was not possible to perform outdoor measurements when it was raining. One participant was not able to participate in outdoor measurements because of a nonstudy-related injury after the laboratory measurements. All the measurements and interviews were conducted by research group members with help from research assistants trained for the task.

### Laboratory Measurements and Interviews

#### Overview

For the indoor research visit, participants were instructed to wear sneakers or running shoes and clothes that were suitable for physical activity, preferably as tight as possible to facilitate the attachment of reflective markers for 3D motion analysis. If the participant did not have shorts or tight leggings, they were provided with tight shorts. Participants were instructed to eat a light meal or breakfast and to avoid alcohol use 24 hours before the measurements.

At the start of the research visit, the MMSE test was conducted and written informed consent was obtained. Participants had the possibility to ask questions about the study and the study protocol was explained to them. After that, weight and height were measured.

#### 10-Meter Walking Test

Indoor walking tests started with a 10-meter walking test to identify participants’ walking speed. The time in seconds was measured using photocells placed 10 meters apart in a hallway. Participants were instructed to walk through the photocells at their usual walking speed, starting 2 meters before the first pair of photocells and stopping 2 meters after the second pair of photocells. The test was done once by each participant. Time was changed into walking speed (in m/s) and this information was used to determine the speed for the subsequent treadmill tests.

#### Next-Generation Inertial Measurement Units

After the 10-meter walking test, next-generation inertial measurement unit (NGIMU) devices (x-io Technologies Limited) were attached. NGIMU is an IMU sensor that includes a triple-axis accelerometer (±16 g; 400 Hz sample rate), triple-axis gyroscope (rotations ±2000°/s; 400 Hz sample rate), and triple-axis magnetometer (magnetic field ±1300 μT). NGIMUs also include analog inputs (8-channel; 0-3.1 V; 10-bit; 1 kHz sampling rate). One NGIMU including housing has dimensions of 56×39×18 mm and weighs 46 g. A total of 8 NGIMU devices were attached to participants with Velcro straps and leukoplast tape.

NGIMUs were placed on both legs above the third metatarsal, on the lateral mid shank, anterior thigh 5 cm above the knee joint, and to the middle of the spine just above the spina iliaca posterior superior and upper half of sternum.

#### VICON 3D Motion Analysis

3D motion capture (Vicon Motion Systems) was used to obtain ground truth values for indoor walking tests. A total of 16 Vicon Vero cameras were positioned around the space where treadmill walking took place. A total of 26 reflective markers were attached to participants with 2-sided tape and secured with leukoplast tape and a self-adhesive bandage (Textbox 1). Data were sampled using Nexus software (Vicon Motion Systems).

**Placement (right and left legs and sides of the body) of the reflective markers for indoor 3D motion analysis.**

- Hallux
  Top of hallux nail
- Toe
  Proximal head of second metatarsal
- Heel
  Heel, at the same level as toe marker
- Ankle
  Lateral malleoli
  Medial malleoli (used only for calibration)
- Shank
  Midway along the shank, laterally (left and right)
- Knee
  Knee extension—flexion axis and lateral side
  Knee extension—flexion axis and medial side
- Thigh
  Midway between KNEE marker (lateral side) and spina iliaca anterior superios (SIAS)
- SIAS
- Spina iliaca posterior superior
- Thoracic vertebrae 10
- Cervical vertebrae 7 (C7)
- Sternum
  Lowest part of the sternum
- Clavicula
  Between the heads of clavicula

NGIMUs and Vicon data were synchronized with an analog trigger, which sent simultaneous pulses to the master NGIMU (400 Hz) and to Nexus software (1000 Hz).

#### Treadmill Walking Tests

##### Level Treadmill Walking

First, a safety harness was put on the participant. Second, the participant walked on the treadmill (Gymstick Walking Pad Pro 44 cm×120 cm) for 1-5 minutes for familiarization. Treadmill speed was gradually increased in increments of 0.5 km/h until the participant’s walking speed from the 10-meter test was achieved or when the participant indicated that he or she did not wish to increase the speed. Participants were allowed to take support from the front and side rails if necessary for balance at any point during assessments, but they were not allowed to hold the rails during the full measurements.

After familiarization, the treadmill was stopped. The actual test started with 5 seconds of standing still in an anatomical posture. When starting, the speed was gradually increased toward the speed obtained from the 10-meter walking test and once achieved, the participant was asked to walk for 3 minutes at that speed. After 3 minutes, the treadmill was slowed down and the participant was asked to stand again for 5 seconds in an anatomical posture.

##### Uphill and Downhill Treadmill Walking

The treadmill was inclined or declined by 5° to correspond with the slope of the outdoor hilly walking tests. The order of test (incline or decline first) was randomly determined by a random number generator. The test speed was determined based on 1-5 minutes of walking at the new gradient, whereby participants were asked to indicate their self-selected speed for uphill or downhill walking. The actual test started with 5 seconds of standing still in an anatomical posture. Then the speed of the treadmill was gradually increased to the test speed, and participants walked for 2 minutes at that speed. After 2 minutes, the treadmill was stopped, and participants again stood for 5 seconds in an anatomical posture.

### Structured Interviews

#### Overview

Following the walking tests, interviews were conducted including structured questions and questionnaires.

#### Demographic Information

Demographic information included age, gender, perceived financial situation (from very poor to very good), highest educational status, living arrangements (living alone, with spouse, or with someone else), type of housing (apartment block, row house, semidetached, or detached house), and time of residence in the current house.

#### Health Condition

Health condition was determined via self-report (5-point scale from good to poor) [23] and by asking about illnesses diagnosed by a doctor from a list of chronic conditions, including different cardiovascular, respiratory, musculoskeletal, and eye diseases, hearing impairment, diabetes, cancer, and incontinence and an open-ended question about any other physician-diagnosed chronic conditions. In addition, participants were asked about whether they had artificial joints and if so, in what joint [23].

#### Functional Vision

Functional vision was assessed using a 7-item vision function questionnaire (VF-7) [24], which is a modified version of the 14-item vision function questionnaire (VF-14) [25]. The VF-7 comprises 7 activities dependent on functional vision and is validated for use in patients with cataracts. Patients are asked how much difficulty they have doing each activity, with or without glasses. The activities are reading small print; seeing steps, stairs, or curbs; reading traffic, street, or store signs; doing fine handwork; cooking; watching television; and driving in darkness. Each question is scored 4, 3, 2, or 1, respectively, if the participant has no, little, moderate, or a great deal of difficulty performing the activity, and 0 if the participant is unable to perform the activity due to poor vision. If a patient does not do an activity for reasons other than his or her vision, the item in question is not included in the scoring. The final score is obtained by averaging responses across all the relevant activities and multiplying by 25. Scores range from 0 (representing maximum impairment) to 100 (representing no impairment).

#### Trail-Making Test

Trail-making test (TMT) was used to measure executive functioning [26]. The TMT consists of 2 parts. Part A involves drawing connective lines between circles that are spread over a sheet of paper and are numbered from 1 to 25. Part B involves drawing connective lines between circles including numbers and letters in order (1-A-2-B-3-C-4-...etc). Participants are asked to draw the lines as fast as possible without lifting the pen from the paper. The researcher pointed out possible errors as they occurred and the participant continued doing the task. The time to complete the task was recorded. The maximum accepted time for part A was 100 seconds, and for part B 240 seconds or 4 mistakes.

#### Depressive Symptoms

Depressive symptoms were assessed with the Center for Epidemiologic Studies Depression scale (CES-D) [27] and balance confidence was assessed with the Activities-specific Balance Confidence scale [28]. History of falls during the previous 12 months was self-reported and separated into injurious and noninjurious falls [29].

#### Outdoor Mobility

##### Life-Space Mobility

Life-space mobility was assessed with the Finnish version of the University of Alabama at Birmingham Study of Aging Life-Space Assessment (LSA) [10,30]. The LSA establishes self-reported movement patterns according to specific life-space levels, ranging from within one's dwelling to beyond one’s town during the 4 weeks preceding the assessment. For each level of life-space (bedroom, home, outside home, neighborhood, town, and beyond town), participants are asked how many days within a week they attained that level and whether they needed help from another person or used assistive devices. A life-space mobility score was calculated (range 0 to 120), reflecting distance, frequency, and independence of movement. Higher scores indicate a larger life-space.

##### Environmental Features

Environmental features near the participant’s home were assessed using “Perceived environmental barriers for outdoor mobility” (PENBOM) questionnaire [31] and with self-evaluation of the home neighborhood environment, including questions about distances to services, closest grocery store, and walking and cycling routes [23].

##### Physical Activity

Self-reported level of physical activity was assessed with a 6-point scale [32] modified from Grimby [33] and Mattiasson-Nilo et al [34]. The levels varied from “1” hardly any activity and mostly sitting to “6” performing competitive sports.

##### Walking Ability and Walking Modifications

Walking ability and walking modifications were evaluated using a standardized questionnaire [35]. The participants were asked whether they had difficulties in walking 2 km with response options (1) able to manage without difficulty, (2) able to manage with some difficulty, (3) able to manage with a great deal of difficulty, (4) able to manage only with the help of another person, and (5) unable to manage even with help. Regarding walking modifications, participants were asked “Have you noticed any of the following changes in walking 2 km?” The response options (yes or no) concerned reduced walking frequency, having given up walking 2 km distances, walking more slowly, and resting while walking the 2 km distance.

#### Physical Performance Tests

Physical performance tests were performed after the interviews. Lower extremity performance was assessed using the short physical performance battery (SPPB) test following the guidelines of the Finnish Institute of Health and Welfare [36,37], which includes 3 subtests, static balance in 3 different positions, 4-meter walking time, and 5 repetitions of sit-to-stand. The balance test was performed without shoes. The walking test was performed at the usual speed and the sit-to-stand test was performed as fast as possible. Both these tests were performed with shoes on. The possible use of a walking aid during the test was also recorded. All subtest results were recorded in seconds and scored from 0 to 4 points, leading to a maximum of 12 points. Higher SPPB test scores indicate better lower extremity performance.

#### Functional Mobility

Functional mobility was assessed with the Timed Up and Go (TUG) test [38]. In the TUG test, the participant stands up from a chair, walks 3 meters, turns around, walks back, and sits on the chair. The TUG test was performed twice at the usual walking speed, with the shoes on. Time in seconds was measured using a stopwatch. Both results were recorded, and the best result (shorter time in seconds) was used. A lower TUG test time indicates better mobility.

Treadmill walking tests took approximately 1.5 hours including attachment of NGIMUs, instructions and walking tests with breaks, after which the participant had a 15-30 minute coffee break. Interviews and physical performance tests took approximately 45-60 minutes per participant. The total duration of the research visit to the sport and exercise laboratory was 2.5-3 hours.

### Outdoor Measurements

#### Overview

Walking tests outdoors were performed approximately 4 days after the laboratory visit. Participants were instructed to wear good walking shoes (trainers, running shoes, etc) and clothes suitable for physical activity. Outdoor tests were conducted only on days with suitable weather conditions (no rain), thus for some participants the time between indoor and outdoor measures was longer than 4 days (range 2-20).

First, participants were equipped with NGIMUs, force-sensitive resistor (FSR)–insoles and a wrist-worn heart rate monitor (Polar Vantage M, Polar, Finland). The same set and placement of NGIMUs were used as in the treadmill walking tests. FSR-insoles were made with two FSRs (18 mm diameter and active area 12.7 mm), which were attached with tape to a thin insole (1-2 mm) and placed under the heel and ball of the foot. The size of the insole was modified according to each participant’s feet and FSR-insoles were inserted in participants’ shoes replacing the insoles they had in their own shoes. The participants reported that they did not notice having the insoles in their shoes, thus the insoles are not likely to affect the gait parameters. The FSRs were connected to a battery-powered open-source Internet of Things development board (M5StickC PLUS, Interlink Electronics) and force data were sampled at 200 Hz via the board’s 12-bit analog-to-digital converter. Internal clocks of the development boards were synchronized before each measurement using an external button connected to the digital pins of the boards. FSR-insoles were connected to a custom mobile phone app via Bluetooth low-energy connection.

The heart rate monitor was placed on the participant’s right wrist. The heart rate was measured to monitor exertion during the outdoor walking tests. A GoPro 9 Hero camera (120 Hz; GoPro Inc) was attached to a modified stroller which was pushed alongside the track. A second GoPro Hero camera was placed at the end of the walking track. NGIMUs and GoPro cameras were synchronized with a trigger device, which sent an analog signal to the master NGIMU and simultaneously turned on a light visible by both cameras. For camera calibration, a 5-second video was filmed of a checkerboard calibration matrix located at the same distance from the camera as the participant’s walking route.

#### A Modified 6-Minute Walking Test

**Figure 2:**
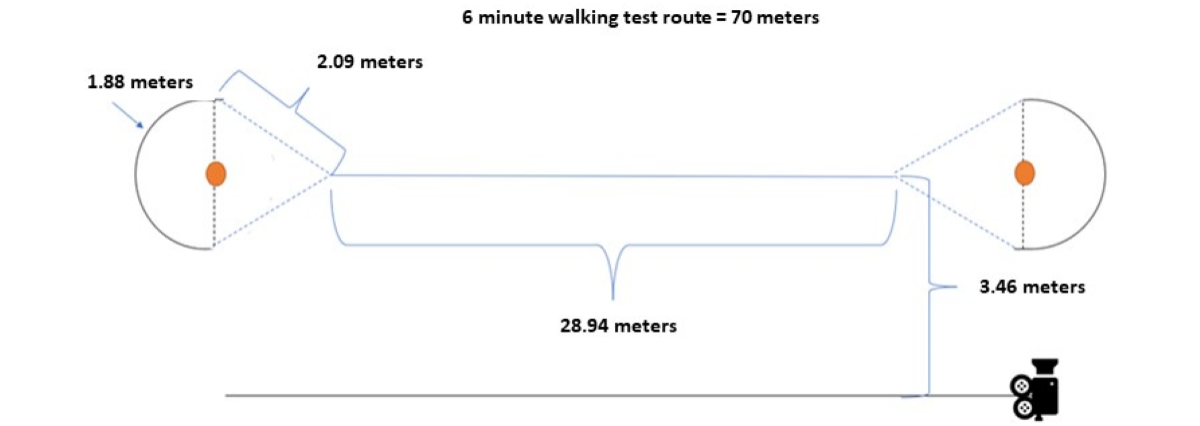
Route of a modified 6-minute walking test performed at a sports track.

Participants walked along a 70 m long route (Figure 2) at the sports track (synthetic track surface) for 6 minutes at a self-selected speed. Rating of perceived exertion was assessed using the Borg scale (range 6-20; 6=no exertion, 20=completely exhausted) [39] at the beginning of the test, after 3 minutes and at the end of the test. A research assistant moved the camera and stroller alongside the participant at a distance of 3.46 meters from the midline of the participant’s route. After 6 minutes of walking, the test was stopped and distance was measured to the nearest meter.

#### Uphill and Downhill Walking Outdoors

A walking test in a hilly environment was performed next to the sports track. The steepness of the hill was 5°, and the ground surface was asphalt.

The participants walked at self-selected speed downhill for 20 meters and then after a short break (approximately 10 seconds) walked 20 meters uphill. Downhill and uphill trials were repeated 5 times, that is, 100 meters walking both ways. A research assistant moved the camera and stroller alongside the participant and a second researcher walked behind the participant for safety. Rating of perceived exertion was assessed after every 20 meters, and the heart rate was recorded with the heart rate monitor. Rest breaks were taken between trials as needed.

### Data Analysis

#### Gait Parameters

Several gait parameters will be derived from IMU data. For example, step duration; step length; stance phase duration; swing phase duration; cadence; gait speed; variability of step, swing, or stance duration; and asymmetry of step, swing, or stance duration.

Step duration is defined as time and step length as the length between 2 consecutive contralateral heel strikes. Stride duration is defined as the time between 2 ipsilateral heel strikes. Swing duration is calculated as the time between consecutive toe-off and heel strike of the same foot, and stance duration as the time between consecutive heel strike and toe-off of the same foot. Cadence is defined as steps per minute and step velocity as step length divided by step duration. Variability in gait parameters (within-person differences in steps) is calculated as SD or coefficient of variation (SD divided by mean multiplied by 100%) of certain parameters per participant.

To calculate gait parameters from different IMUs we will use algorithms published in previous literature (eg, [40-43]). New algorithms are developed when necessary.

#### Vicon Data

Exported 3 dimension motion data are analyzed with open-source Mokka software (Motion kinematic & Kinetic analyzes, biomechanical tool kit, GitHub), where heel strikes and toe-offs are manually determined. In treadmill walking, heel strikes are set to the last frame before the heel marker starts to move backward in the image, that is, at the most anterior point. Toe-offs are set to the last frame before the hallux marker starts to move proximally, that is, at the most posterior point in the image. These analyses are performed by 2 researchers and similarity of identifications is ensured by checking agreement on data. In case of disagreements, event timings are discussed and agreed upon together. Events are then exported to Matlab (b2022; The MathWorks, Inc).

#### GoPro Camera Data

The GoPro camera data were used as a reference to validate the IMU’s in the outdoor environment. From outdoor video data, heel strike and toe-off events and step length are digitized and identified manually. The heel strike is denoted as the first frame when the shoe touches the ground after the swing phase. Toe-off is determined as the last frame when the distal end of the shoe is still on the ground. These analyses are performed by 2 researchers and similarity of identifications is ensured by checking agreement on data. In case of disagreements, event timings are discussed and agreed upon together. ShotCut software (Meltytech, LLC) is used to mark event timings, which are copied to an Excel file (Microsoft Corporation). Timings are converted from frames to seconds using Matlab (MathWorks) and Excel.

### Statistical Analyses

To test the validity of IMU sensors, we will use Bland-Altman plots and intra-class correlation coefficients. To compare gait parameters in different environments, we will use appropriate statistical methods depending on data properties, which may include *t* tests and repeated measures ANOVA and mixed effect models. Linear regression analyses are used to study associations with life-space mobility and physical activity. Other statistical testing will be performed where appropriate.

### Data Management

All data are handled and registered according to the Finnish Personal Data Act and the European Union Data Protection Act. All data are recorded and analyzed without direct personal recognition information. The research material is carefully maintained, documented, and stored in password-protected organizational servers.

### Ethical Considerations

The study was conducted according to good scientific and clinical practices as laid down by the Declaration of Helsinki. All participants were informed carefully about the study, and they gave their written informed consent prior to any measurements. Data collection included no invasive or potentially physically or psychologically harmful elements beyond what one might experience in everyday life. Only people who were personally able to consent were recruited. Research assistants conducting the data collection were trained for study procedures and the safety of participants during the measurements was ensured. Participants had the possibility to withdraw from the research at any point without any consequences. The ethics committee of the JAMK University of Applied Sciences approved the study (JAMK/40/13.02/2021; January 11, 2021).

## Results

The GaitAge data collection is completed. A total of 40 participants participated in indoor measurements and 39 of them in outdoor measurements. Participants were on average 76.3 (range 69-92, SD 5.45) years of age, 65% (n=26) of them were women, and all of them reported being able to walk 2 km without difficulties.

Gait data analyses are underway, and the first results reporting findings of the validation of NGIMUs is expected to be published in spring 2024.

## Discussion

### Principal Findings

Our project features several strengths. First, for example, a comparison of gait parameters in an outdoor environment and on a treadmill in a laboratory provides fundamental knowledge for studies aiming to use gait parameters to predict progressive neurological problems. We will report how older adults adapt their gait according to variable environmental demands. This study provides findings that can change our view of person-environment interaction processes by providing detailed information about changes in gait parameters in level, uphill, and downhill walking. Second, we will assess whether changes in gait biomechanics induced by environmental demands influence possibilities for general participation in outdoor activities of older people by combining information on gait parameters with life-space mobility. Third, we will explicitly focus on person-environment interaction at the individual level, which will create new study hypotheses and give new perspectives on research in environmental gerontology. Fourth, the study sample of 40 older adults with a comprehensive data set (several walking measures with IMUs, 3D motion analyses and video data, questionnaires, and physical performance tests) will have a multidisciplinary impact by providing information about objective and subjective aspects of gait and mobility in old age.

It should be noted that the study population included older adults without major mobility difficulties or neurological conditions, so the results cannot be generalized to clinical populations. There are also some limitations in the study protocol that will be taken into consideration in forthcoming analyses, for example, differences between measurement protocols for level treadmill walking (3 minutes) and outdoor walking (6 minutes). We will also need additional longitudinal studies to determine whether possible changes in gait in different environments are predictive of changes in mobility capacity.

### Conclusions

This research will provide answers to major research questions about the role of the environment in outdoor mobility in old age. This research project could serve as a reference for future research on gait among different patient groups and will serve as a baseline for a longitudinal study, with the aim of exploring whether changes in gait parameters in different environments can be used as early signs of declining health and restricted participation in outdoor activities.

## Abbreviations

CES-D: Center for Epidemiologic Studies Depression scale
FSR: force-sensitive resistor
IMU: inertial measurement unit
LSA: Life-Space Assessment
MMSE: Mini-Mental State Examination
NGIMU: next-generation inertial measurement unit
PENBOM: perceived environmental barriers for outdoor mobility
SPPB: short physical performance battery
TMT: trail-making test
TUG: Timed Up and Go
VF-7: 7-item vision function questionnaire
VF-14: 14-item vision function questionnaire

## Data Availability

The data sets generated and analyzed during this study are available from the corresponding author (MR) upon reasonable request.
